# mmWave High Gain Planar H-Shaped Shorted Ring Antenna Array

**DOI:** 10.3390/s20185168

**Published:** 2020-09-10

**Authors:** Young-Jun Kim, Ye-Bon Kim, Han Lim Lee

**Affiliations:** School of Electrical and Electronics Engineering, Chung-Ang University, 84 Heukseok-ro, Dongjak-gu, Seoul 06974, Korea; yhjkim747@cau.ac.kr (Y.-J.K.); equa1ity@cau.ac.kr (Y.-B.K.)

**Keywords:** mmWave antenna array, high gain antenna, slot-loaded antenna, antenna matching

## Abstract

A new design approach for a mmWave high gain planar antenna is presented. The proposed method can increase antenna directivity with a minimally enlarged radiation patch while the operation frequency is still matched at a higher target frequency. The fundamental structure of the proposed antenna is configured by a H-shaped and slot-loaded patch with a shorting pin symmetrically located across a signal excitation port. Further, to match the operation frequency with the frequency for the highest achievable gain, a vertically stacked matching conductor was inserted along the signal feed path between the radiation patch and the ground layer. The proposed single antenna showed the simulated directivity of 9.46 dBi while the conventional patch with a same dielectric had 8.07 dBi. To verify practical performance, a 2 × 2 array antenna was fabricated at 28 GHz and showed the measured gain of 12.5 dBi including the array feed loss.

## 1. Introduction

In order to meet the increasing demand for faster and reliable communication in both 5G and beyond 5G (B5G), mmWave spectrum is being actively investigated to fully accommodate the rapid growth in expected data traffic. Further, the advent of 5G communication has prompted the intensive research for Internet of Things (IoT) devices converged with 5G network, as well. Thus, in order to deploy a successful and reliable next generation services, the improvement in mmWave technologies must occur. Although there is a broad spectrum of research to solve unprecedented challenges in mmWave, antenna technology should be a primary concern as a massive number of antenna elements are required. To overcome the high propagation path loss at mmWave, phased array antenna is leading the main technical stream. Further, since high gain antenna elements can eventually reduce the required number of both antenna elements and mmWave beamforming integrated circuits (ICs) in an array configuration, the overall antenna module or system size, number of required parts, cost, fabrication difficulty and power consumption are effectively reduced. Although the conventional patch antennas are typically used due easy integration with multiple beamforming ICs, the demand for a planar antenna structure with higher gain is increasing to accommodate the aforementioned advantages in high gain antennas. Previously, research for high gain antenna structures has been extensive. Some previously reported antennas adopt cavity or slot-loaded structures to achieve high gain [1,2,3], but the sacrifice of high-profile cavity or impractical geometry make them unsuitable for mmWave applications. Other structures based on periodic or Yagi-like structures, or dielectric resonators were also reported [4,5,6,7], but they still require a large dimension as well as a high profile. Additionally, stacked structures based on planar patch radiators have been proposed [8,9], but the gain enhancement ratio with respect to the increase in overall volume, including the stack height and ground plane size, seems to be less effective. Further, a planar magneto-electric dipole antenna with high directivity was recently reported [10]. However, the required dimension is still large and the precise use of multiple via increases the difficulty in both fabrication and performance tuning. In addition, metasurface, cavity backed, cavity resonance antennas are considered for high gain array antenna [11,12,13], but they require a high profile or a large occupation of extra layers limiting the integration with beamforming ICs. Lastly, complementary dipole and slot array structure, and phased array quasi-Yagi antenna have been proposed [14,15], but the high profile and complicated coupling aperture feed network seems to be the bottleneck.

Thus, to overcome the drawbacks of the previously reported structures, a new design configuration for mmWave high gain structure without using a superstrate configuration is proposed. Further, a practical mmWave antenna should be configured in an array or a sub-array architecture with an integrated feed network. However, when multiple antenna elements are integrated in an array configuration, the amplitude and phase balances among the radiation elements usually become unmatched and differ from each other within an array, requiring a compensation technique. Thus, the proposed single element is extended to a 2 × 2 array structure including the feed network for ensuring the amplitude and phase balances among radiation elements and verified at 28 GHz.

## 2. Analysis of the Proposed Antenna Array

**Figure 1:**
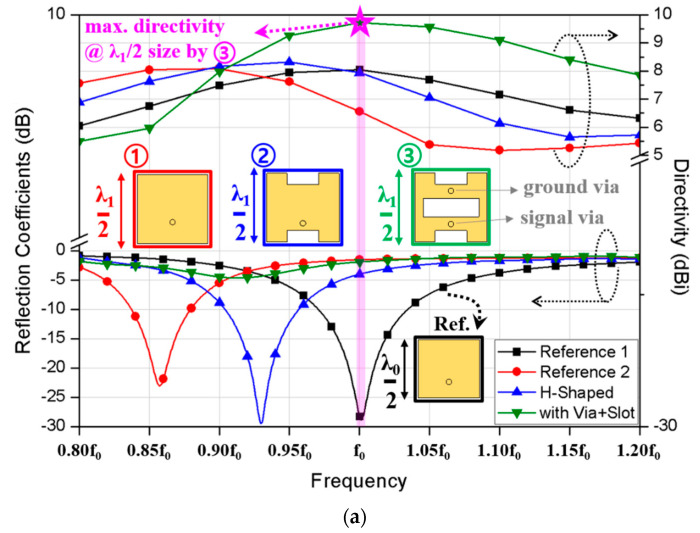

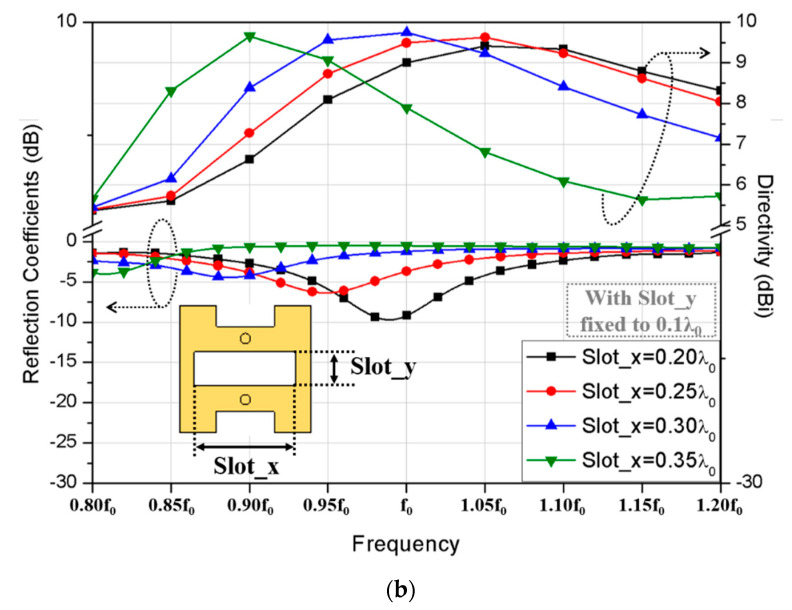
Reflection coefficient and directivity behaviors for (**a**) different types of planar patches and (**b**) a proposed slot-loaded patch with a shorting pin.

Microstrip patch antenna is usually preferred due to its simple architecture, low-complex design, ease of fabrication and integration with ICs, and good performance [16,17]. The conventional patch antenna is designed by a half wavelength at its operation frequency as shown by the reference 1 antenna in Figure 1a. Here, 3-D simulation extracted data by CST studio suite software are used to present the plots. The reference 1 antenna operates at f_0_ denoting a desired target operation frequency. It is known that the maximum achievable gain of antenna theoretically depends on its physical size compared to the wavelength at an operation frequency. However, if the antenna size is simply enlarged to λ_1_/2 to increase the potential gain, the operation frequency lowers due to the increase in wavelength without a gain enhancement at the center frequency. That is, although the increase in antenna size can increase the potential gain or directivity, the shift in matched frequency is unavoidable due to the change in a wavelength.

As shown in Figure 1a, the reference 1 and 2 of the conventional patches with the center frequencies at f_0_ and f_1_, where f_0_ is the target frequency higher than f_1_. Reference 2 shows the corresponding patch size to λ_1_/2 and the maximum potential directivity of 8 dBi at its matched frequency. This directivity is similar to the potential directivity of the reference 1 conventionally designed at the target frequency. Instead of using the reference 1 directly, the reference 2 with a physically larger dimension is used at the target frequency by shifting the matching frequency through H-shape as shown in Figure 1a. Although the frequency shifts up slightly, the maximum gain still remains similar to the gain at lower frequency. Then, additional slot is integrated with a shorting pin to control resonant current that is shown in a green box inside Figure 1a. Since additional field radiated through the slot from the ground conductor is converged with the radiated field from the shorted patch, the maximum achievable directivity is increased by approximately 2 dB within the same dimension. However, the frequency is unmatched even though the maximum gain is achieved at the target frequency. Thus, to match the operation frequency while keeping the maximum directivity, the slot size is adjusted as shown in Figure 1b. Since the variation in slot y dimension has not affected the electrical characteristics significantly, and the slot x dimension is varied with the fixed slot y. However, within the physically available size of the patch, the changes in slot dimension cannot satisfy both the impedance matching and highest directivity simultaneously at the desired frequency. Therefore, to overcome the limitation in the physically enlarged antenna with a simple slot or a shorting pin, a new H-shaped shorted ring configuration with a vertically loaded stack feed is proposed.

**Figure 2:**
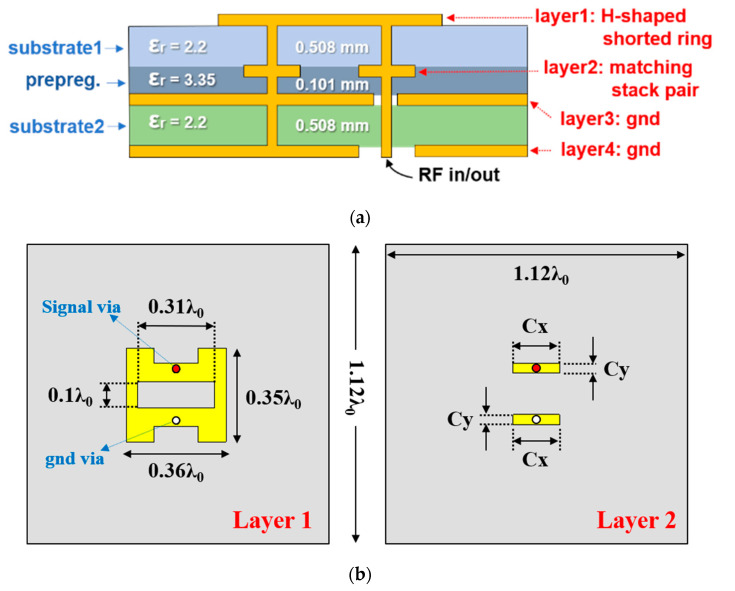
Proposed antenna structure with (**a**) multi-layer information and (**b**) layer 1 and layer 2.

Figure 2 shows the proposed H-shaped shorted ring with a pair of vertically loaded conductor stacks along the signal feed and shorting pin. The proposed structure is designed with 4-layers using two substrates and a single prepreg as shown in Figure 2a. The slot-loaded patch with a shorting pin is designed on layer 1 and additional matching stack is inserted into layer 2. The matching stack consists of a pair of rectangular conductors where one is loaded on the signal feed line and the other is connected to the ground. Figure 2b shows the dimension and matching stack parameters. 

**Figure 3:**
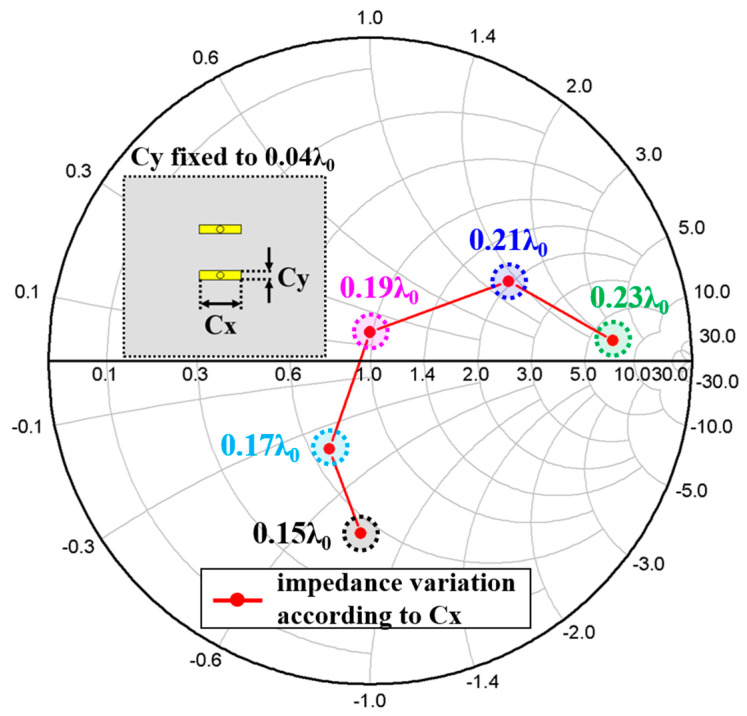
Proposed antenna structure with impedance variation according to Cx value.

Depending on the values of Cx and Cy, loaded inductance and capacitance are varied. Since the increase in Cy heavily loads the capacitance on the antenna, Cy value is minimized to the size corresponding to the through-via size. Having the Cy fixed to 0.04 λ_0_, the effect of variation in Cx value on the antenna impedance is simulated as shown in Figure 3. Starting from the arbitrary chosen value of 0.15 λ_0_, the increase in Cx shows the additional inductive and capacitive loading that makes the impedance match to the operation frequency by Cx of 0.19 λ_0_. Here, the λ_0_ at 28 GHz corresponds about 10.7 mm and the Figure 3 represents the parameter variation according to 0.2 mm in length. Since the typical printed circuit board (PCB) technology where a fabrication resolution of 0.1 mm can be guaranteed, the concern about fabrication sensitivity of the proposed architecture can be negligible.

**Figure 4:**
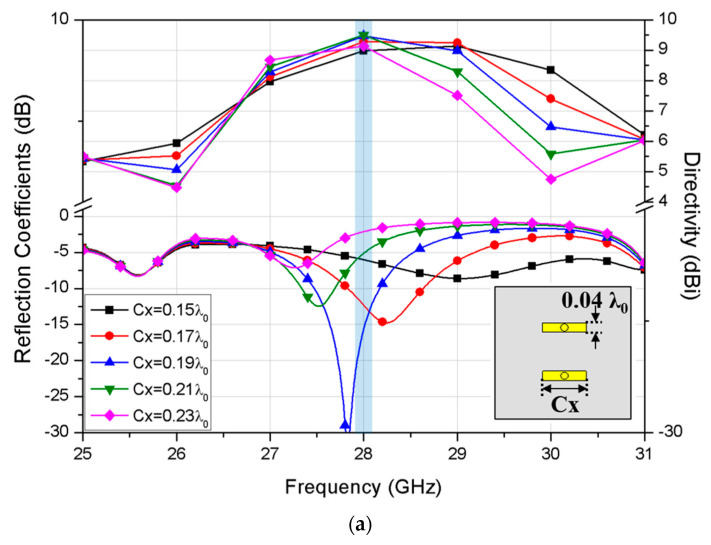

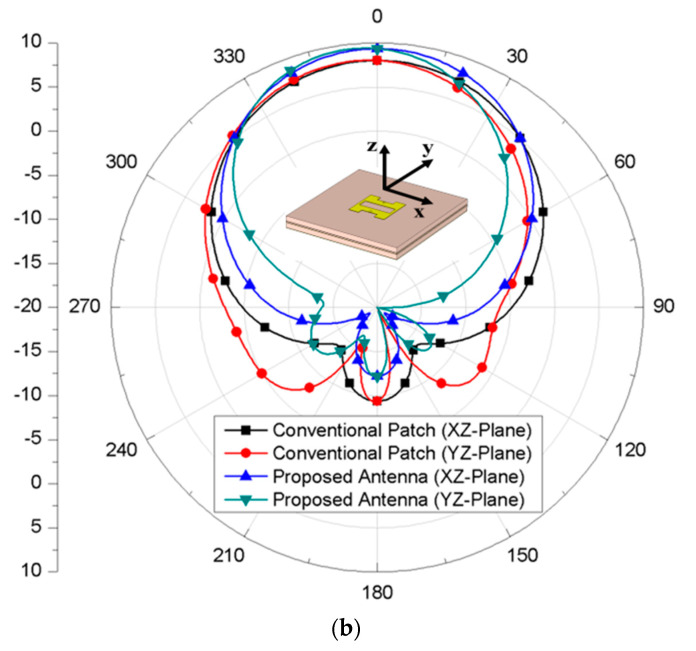
Simulated results for (**a**) reflection coefficient and maximum directivity according to Cx values, and (**b**) radiation patterns of the proposed and conventional patch antennas.

Further, the effect of variation in Cx value on the achievable directivity is also simulated as shown in Figure 4a. Having the Cx and Cy set to 0.19 λ_0_ and 0.04 λ_0_, respectively, it confirms that simultaneous matching for reflection coefficient and maximum directivity were achieved at the target frequency of 28 GHz. Then, radiation pattern of the proposed antenna is simulated and compared with the conventional patch antenna with the same ground size as shown in Figure 4b.

The simulated peak gains of the proposed antenna and the conventional patch are 9.46 dBi and 8.07 dBi, respectively, showing the gain enhancement by 1.39 dB. Also, the half power beamwidth (HPBW) of the proposed antenna shows 67° and 55° in xz and yz planes, respectively, while the simulated radiation efficiency is about 91.34%.

**Figure 5:**
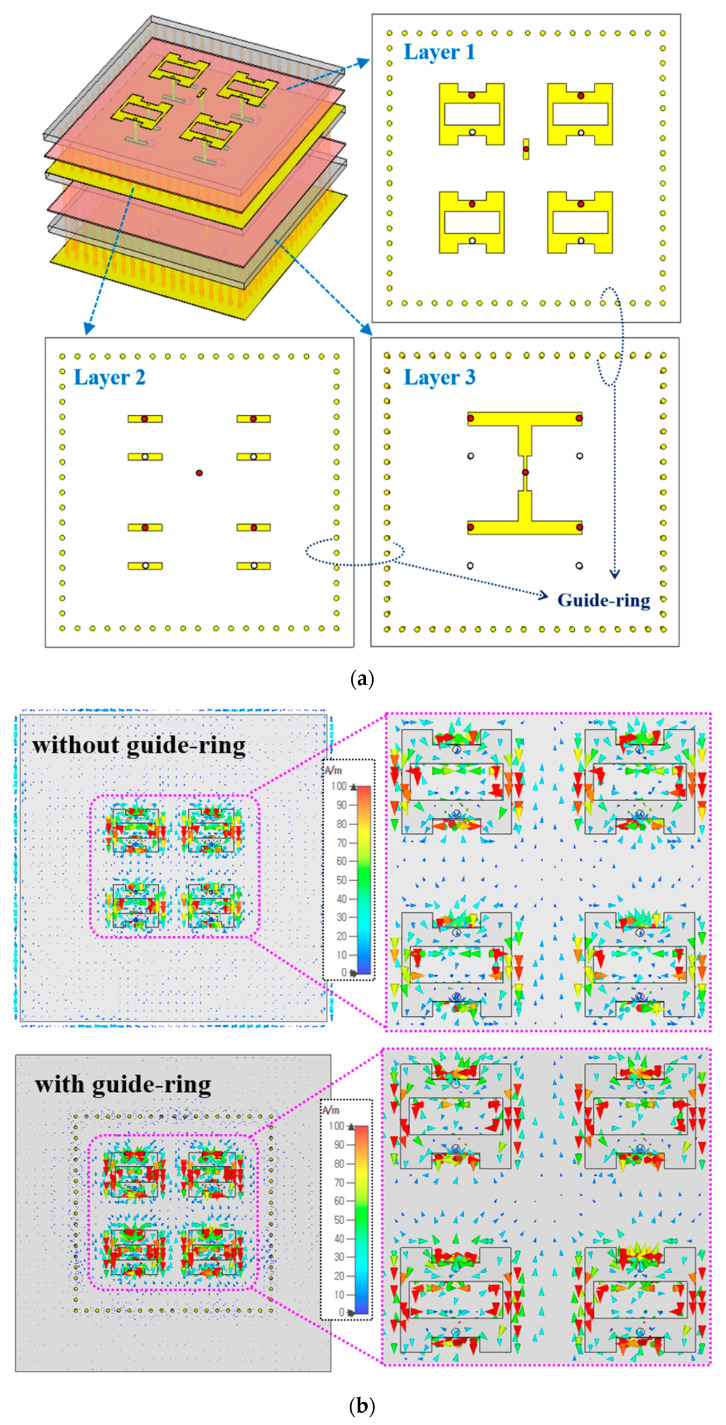
Proposed 2 × 2 array antenna with (**a**) multi-layer view and (**b**) surface current on array elements synchronization by a guide-ring.

To validate the effective use in a practical array configuration, the proposed antenna structure is extended to the 2 × 2 array as shown in Figure 5a. The same substrates and prepreg materials as described in Figure 2a are used and the four slot-loaded radiation elements with shorting pins are integrated on the top layer. Also, each antenna element is vertically loaded with a pair matching stack as shown in layer 2 while the 1 × 4 feed network is integrated into the layer 3 to excite the four radiation elements with a single RF port. Further, an additional guide-ring of 1.55 λ_0_ × 1.54 λ_0_ as a magnetic wall is integrated with the proposed array antenna. Since mmWave antennas are relatively small compared to RF connectors used for measurement, a large ground plane is usually required to fabricate the mmWave antenna sample. However, the large ground plane causes the unnecessary spread of RF currents, resulting in a shift of matching frequency or gain degradation. Further, in the array structure, a phase mismatch in surface current of each radiation element occurs without the guide-ring as shown in Figure 5b. The upper two elements and lower two elements have different current distribution due to the phase mismatch. Thus, by integrating the guide-ring to prevent the RF current from spreading and keeping the surface currents among each element uniformly excited, the antenna gain can be further optimized.

## 3. Fabrication of the Proposed Antenna and Measured Results

**Figure 6:**
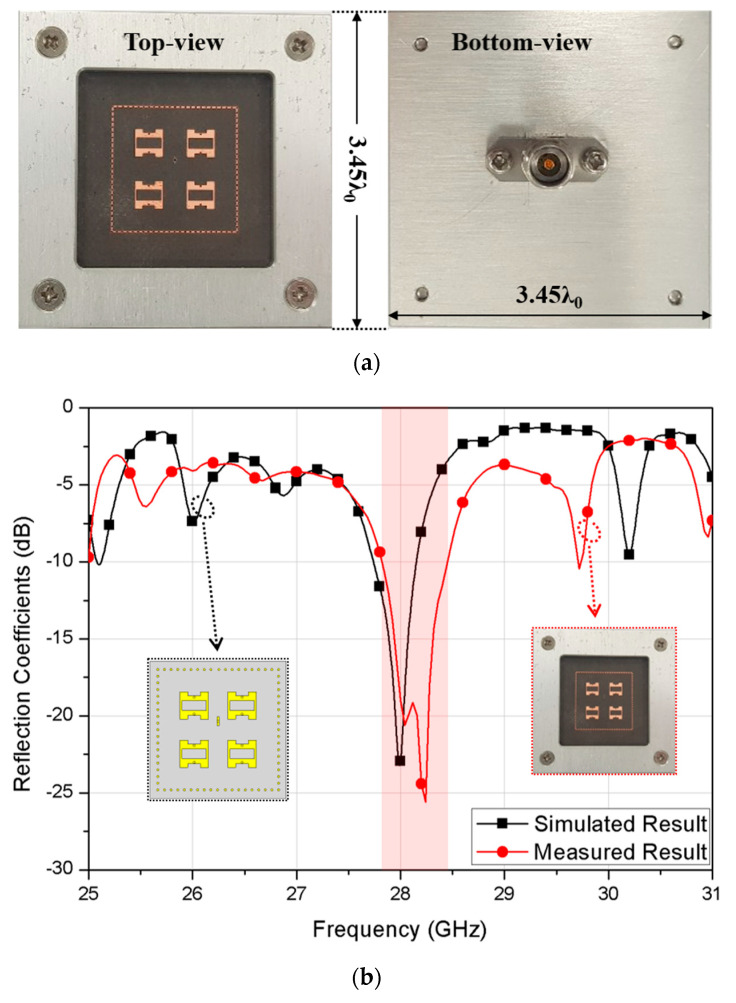
Implementation of the proposed 2 × 2 array antenna with (**a**) fabrication photo and (**b**) simulated and measured reflection coefficients.

The proposed 2 × 2 array antenna was fabricated with Rogers 5880 substrate with a relative permittivity of 2.2 and loss tangent of 0.0009, and Rogers prepreg RO4450T with a relative permittivity of 3.35 and loss tangent of 0.004 as shown in Figure 6a. Also, an aluminum test jig was assembled with the fabricated antenna array to tightly fix the RF connector for stable measurement.

**Figure 7:**
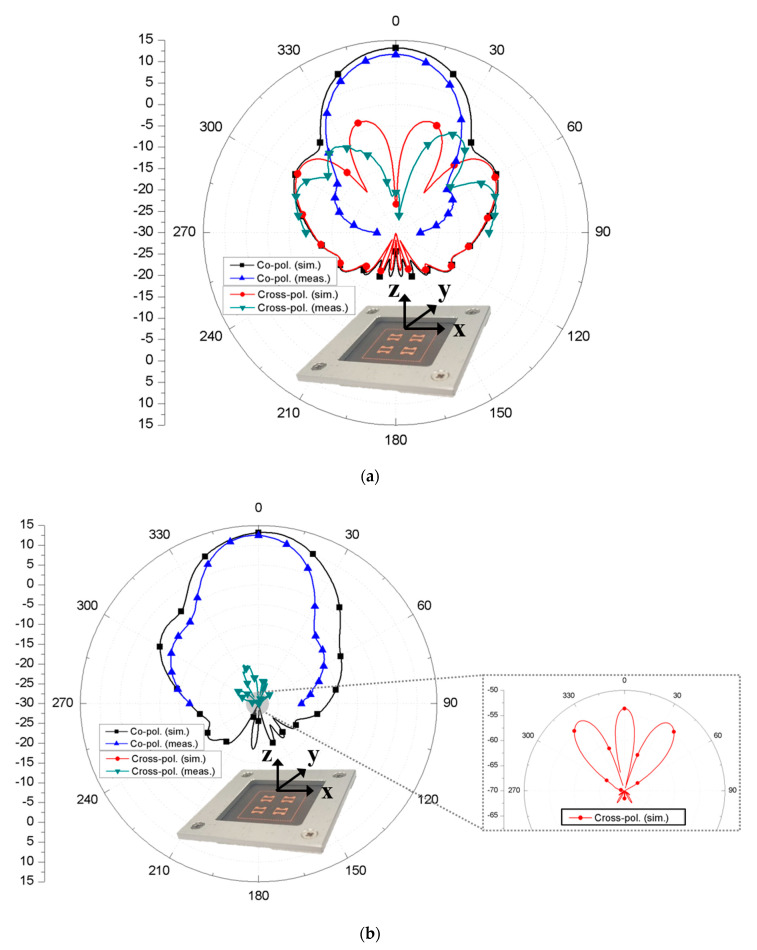
Simulated and measured radiation patterns of the proposed 2 × 2 array antenna in (**a**) xz-plane and (**b**) yz-plane at 28 GHz.

The fabricated volume of the fabricated antenna was 3.45 λ_0_ × 3.45 λ_0_ × 0.17 λ_0_ at 28 GHz including the whole ground plane size while the guide-ring area occupied about 1.55 λ_0_ × 1.54 λ_0_. The simulated and measured 10-dB impedance bandwidth of the proposed 2 × 2 array antenna were 1.43% and 2.29%, respectively. Here, the measured bandwidth was slightly increased possibly due to the effect of the test jig required for fixing the RF connector firmly. Having the proper fixture is critical in mmWave antenna measurement since it provides consistent measured results. Thus, the measured result is reasonable in comparison with the simulated result. Finally, the simulated and measured radiation patterns of the proposed antenna at 28 GHz are shown in Figure 7a,b. The simulated and measured peak gains were 13.2 dBi and 12.5 dBi, respectively, including the 1 × 4 feed loss. We noted that the decreased gain is suspected due to the antenna test jig. This result also implies that the increased bandwidth is the result of more loss added to the return loss. Further, the measured HPBWs in xz and yz planes of the proposed antenna were about 34° and 31°, respectively, while the simulated HPBWs were about 36° and 37°, respectively. The measured minimum cross-polarization discrimination (XPD) level showed more than 15.5 dB. Therefore, the feasibility of the proposed mmWave antenna structure was verified by the good agreement between the simulated and measured results at 28 GHz. Lastly, the performance comparison with the other antenna topologies is summarized in Table 1.

## 4. Conclusions

Owing to the increased number of antenna elements to support advanced beamforming technology or next generation massive MIMO communication, effective antenna structure with a high directivity that potentially lowers the mmWave RF system burden must be required. Thus, a new antenna structure satisfying both enhanced gain and matched operation frequency simultaneously has been proposed in this communication. The antenna was configured by a planar slot-loaded patch with a shorting pin where a signal feed line was vertically loaded with a matching stack. The proposed antenna was analyzed in both single and 2 × 2 array configurations and verified at 28 GHz. Further, the feed network based on T-junction with guide-ring was integrated within the array configuration to maximize the array gain. Finally, the measured result showed the high directivity of 12.5 dBi including the loss by array feed network.

## Figures and Tables

**Table 1:** Comparison of the proposed antenna performance with the other antenna topologies.

Reference	Polarization	Topology	Cent. Freq. (GHz)	BW (%)	Gain (dBi)
[2]	Linear (slant)	Cavity-backed	10.1	1.0	6.9
[3]	Circular	Slot	5.2	49.8	8.5
[4]	Linear	I-Shaped resonator	7.8	47.7	9.5
[5]	Linear	Slot	4.2	27.8	12.2
[6]	Linear	H-shaped resonator	3	51.9	9.7
[7]	Linear	Dielectric resonator	15	16.1	10.4
[8]	Linear	Stacked	5.73	34.9	8.07
[9]	Linear	Slot + stacked	2.4	19.6	9.7
[10]	Linear	Via-loaded	10.3	9.1	10.2
[11]	Linear	Metasurface	5.6	2.5	12.2
[12]	Linear	Cavity-backed	11.7	21.4	10.0 *
[15]	Linear	Qausi-yagi	26	7.7	8.31 **
This work	Linear	Shorted ring + slot	28	2.3	12.5

* Measured gain for 2 × 2 array with feed loss; ** Simulated gain for 2 × 2 array without feed loss.
